# Mass coral mortality under local amplification of 2 °C ocean warming

**DOI:** 10.1038/srep44586

**Published:** 2017-03-23

**Authors:** Thomas M. DeCarlo, Anne L. Cohen, George T. F. Wong, Kristen A. Davis, Pat Lohmann, Keryea Soong

**Affiliations:** 1 Massachusetts Institute of Technology/Woods Hole Oceanographic Institution Joint Program in Oceanography/Applied Ocean Science and Engineering, Woods Hole Oceanographic Institution, Woods Hole, MA 02543, USA; 2 Woods Hole Oceanographic Institution, Woods Hole, MA 02543, USA; 3 Research Center for Environmental Changes, Academia Sinica, 128 Academia Road Section 2, Nankang, Taipei 115, ROC, Taiwan; 4 Old Dominion University, Norfolk, VA, USA; 5 University of California, Irvine, California, USA; 6 National Sun Yat-sen University, Kaohsiung, ROC, Taiwan

## Abstract

A 2 °C increase in global temperature above pre-industrial levels is considered a reasonable target for avoiding the most devastating impacts of anthropogenic climate change. In June 2015, sea surface temperature (SST) of the South China Sea (SCS) increased by 2 °C in response to the developing Pacific El Niño. On its own, this moderate, short-lived warming was unlikely to cause widespread damage to coral reefs in the region, and the coral reef “Bleaching Alert” alarm was not raised. However, on Dongsha Atoll, in the northern SCS, unusually weak winds created low-flow conditions that amplified the 2 °C basin-scale anomaly. Water temperatures on the reef flat, normally indistinguishable from open-ocean SST, exceeded 6 °C above normal summertime levels. Mass coral bleaching quickly ensued, killing 40% of the resident coral community in an event unprecedented in at least the past 40 years. Our findings highlight the risks of 2 °C ocean warming to coral reef ecosystems when global and local processes align to drive intense heating, with devastating consequences.

An historic international agreement reached at the Conference of Parties 21^st^ session (COP21) aims to limit global warming within 2 °C above pre-industrial levels by reducing greenhouse gas emissions, an attempt to avert the most devastating impacts of climate change^1^. Yet there is growing concern that the 2 °C limit is wholly insufficient to protect the world’s at-risk populations and ecosystems^2^,^3^. Tropical coral reefs, which provide hundreds of millions of people worldwide with food and income, fall in this category^4^,^5^,^6^. Considered some of the ocean’s most productive ecosystems, coral reefs are particularly sensitive to ocean warming, which disrupts the symbiotic relationship between coral animals and their photosynthetic algae. Bleaching, so-called because corals turn white as they expel algae, is lethal if prolonged^7^. In the past 30 years, coral bleaching caused by ocean warming has contributed to the loss of 19% of the world’s coral reef area^8^,^9^.

Global climate model simulations of open-ocean warming under business-as-usual greenhouse gas emissions scenarios imply that bleaching will occur annually on over 80% of the world’s remaining reefs by the second half of this century^10^,^11^. Strategies designed to cap ocean warming have potential to reduce or at least delay these impacts^10^,^11^, and some corals may be able to adapt to moderate rates of warming^10^,^12^. Critically however, the thermal environments of many tropical coral reef ecosystems do not resemble those of the open ocean^13^,^14^,^15^,^16^,^17^,^18^,^19^. Limited by availability of light, tropical reefs form relatively shallow structures, even those perched atop undersea volcanoes whose bases stretch kilometers to the seafloor. In these shallow environments, regional weather conditions and local hydrodynamics exert strong influence and when they align, a moderate open-ocean warming can quickly translate to intense heating on the reef, with devastating consequences.

Here we document the physical and ecological consequences of such an alignment on Dongsha Atoll, a coral reef in the northern South China Sea (SCS). We developed a reef-flat heat budget to diagnose the physical drivers of warming in June 2015, linking it to a regional atmospheric anomaly, and tracked the coral community response to extreme thermal stress. Finally, we evaluate the historical precedence for an event of this magnitude using markers of thermal stress preserved in the skeletons of long-lived corals.

## Results and Discussion

**Figure 1:**
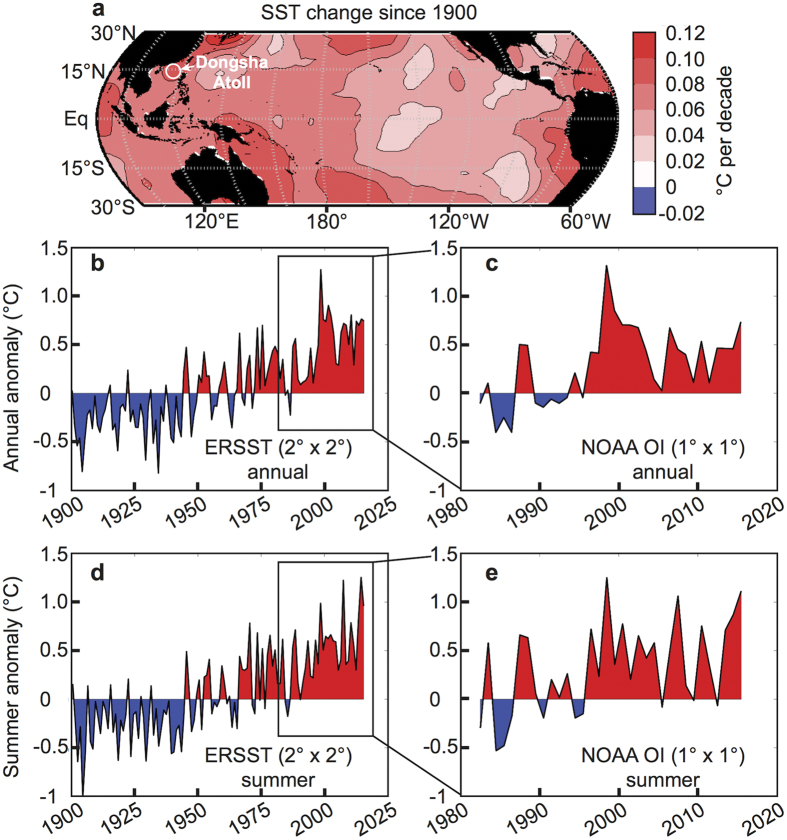
Historical sea surface temperature (SST) changes. (**a**) Map of the rate of SST increase since 1900 (data from Extended Reconstructed Sea Surface Temperature, ERSST). The northern SCS has warmed at a rate similar to other coral reef regions such as Papua New Guinea, Melanesia, and the eastern Caribbean. (**b**,**c**) Annual mean and (**d**,**e**) summer (June-July-August, or JJA) SST anomalies for the open-ocean surrounding Dongsha Atoll. The data are plotted in (**b** and **d**) for 1900–2015 from ERSST and in **c** and **e** for 1982–2015 from NOAA Optimal Interpolation (OI). In all panels, the data are derived from the single gridbox (2° resolution for ERRST and 1 °C resolution for OI) covering Dongsha Atoll. Red and blue shading corresponds to years that were warmer or cooler, respectively, than the 1940–1970 climatology calculated from ERSST. Anomalously warm SST often occurred in the South China Sea during strong El Niño events, especially pronounced during summertime in 1998, 2007, and 2015. The map in panel **a** was created with MATLAB2012a (http://www.mathworks.com/) using data sources described in the Methods.

**Figure 2:**
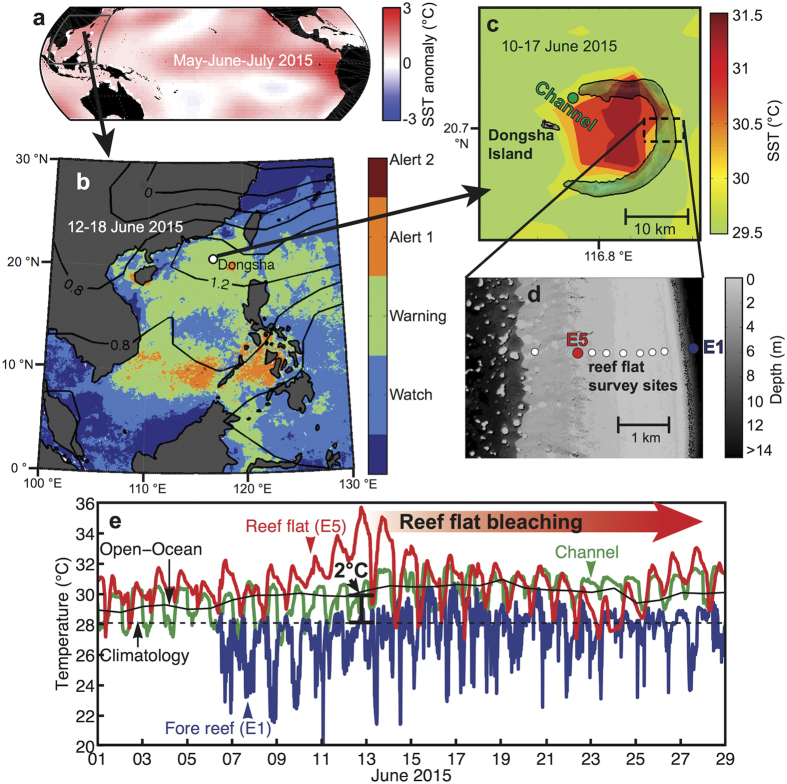
Local amplification of regional warming. (**a**) May-June-July (MJJ) 2015 SST anomalies relative to the 1940–1970 MJJ ERSST climatology shows Pacific basin-wide warming in response to developing El Niño conditions. (**b**) NOAA Coral Reef Watch 7-day maximum alert level (colours) for the South China Sea region 12–18 June 2015. Black contours are June 2015 monthly sea level pressure anomaly (hPa) from NCEP/NCAR reanalysis, showing a high-pressure system centered over Dongsha Atoll (white circle). (**c**) SST in and around Dongsha Atoll 10–17 June 2015 derived from 4-km MODIS data. SST is higher within the lagoon and on the submerged reef flat compared to the adjacent open-ocean. Dashed black box is where ecological surveys were conducted. (**d**) Bathymetry map of the eastern reef flat (courtesy of Dongsha Atoll research station) highlights the shallow habitat created by the atoll. (**e**) Temperatures captured by *in situ* loggers on the reef flat (red), in the channel (green), and on the fore reef (blue); and open-ocean (solid black) and the open-ocean climatological mean for June (dashed black; NOAA Coral Reef Watch 5-km product). Open-ocean temperatures were 2 °C above normal (black bar) in mid-June but were amplified to 6 °C on the shallow reef flat, triggering mass bleaching and mortality. The maps in panels (a–d) were created with MATLAB2012a (http://www.mathworks.com/) using temperature data sources described in the Methods. The Dongsha Atoll Research Station supplied the outline of Dongsha Atoll in panel **c** and the reef flat bathymetry data in panel (d).

The ocean surface surrounding coral reefs in the northern SCS has warmed at a rate of 0.09 ± 0.015 °C/decade (95% confidence) since 1900 (Fig. 1), closely tracking rates of SST rise globally (0.07 °C/decade)^20^,^21^, in the tropics (0.08 °C/decade)^22^, and in coral reef regions (0.09 °C/decade)^22^ (see also Fig. 1a and refs 14,22, 23, 24 for spatial variability). In spring and summer 2015, weakened surface winds in the northern SCS associated with a developing Pacific El Niño and diminished sea to air latent heat flux^25^,^26^,^27^, were superposed on this secular trend (Fig. 1d,e). The resulting warming of the sea surface culminated in a June SST anomaly 2 °C above the expected temperature for that time of year (Fig. 2e). Nevertheless, the open-ocean SST anomaly was not high enough for long enough to raise NOAA’s Coral Reef Watch “Bleaching Alert” (Fig. 2b), and coral bleaching was not anticipated on reefs in the northern SCS in June 2015 (ref. 28).

**Figure 3:**
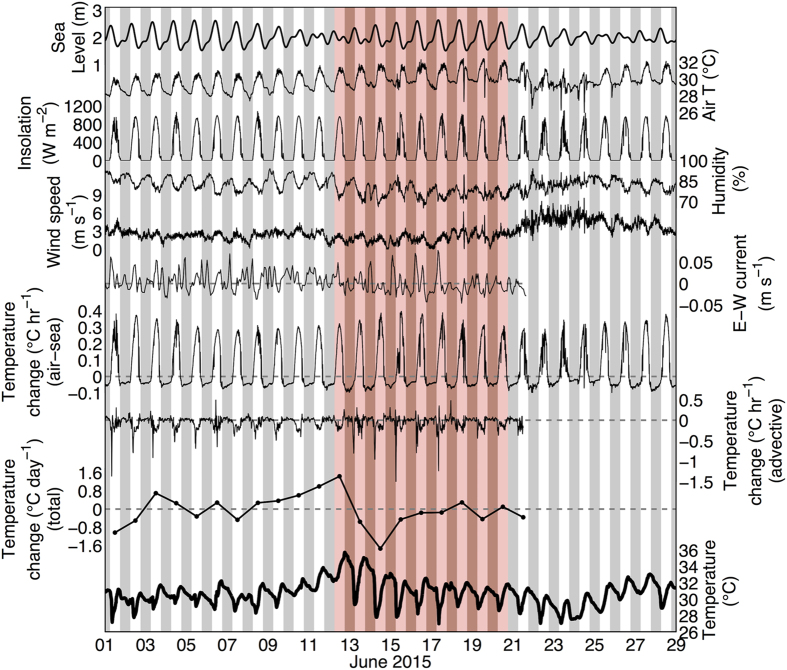
Reef flat heat budget and local physical conditions leading to bleaching. Time series of weather conditions on Dongsha Island, current speeds, estimated heat budget components, and measured water temperature on the reef flat. White background is daytime, shaded background is nighttime, and red-shaded background indicates the time within which the onset of bleaching is constrained by visual observations on the reef flat (*i.e.* no bleaching observed on 12 June, and all corals visually bleached on 20 June). From the beginning of June 2015 to the onset of bleaching in mid-June, relatively high air temperatures, insolation, and humidity; and low wind speeds created favorable conditions for high air-to-sea heat flux. However, while water temperatures increased >4 °C between early and mid-June, the calculated air-sea heat flux maintained a consistent diurnal cycle of nearly constant amplitude, suggesting that the advective component of the heat budget was responsible for the warming between early and mid-June. Indeed, the calculated advective heat flux shows large cooling events that occur daily, but begin to diminish in strength and nearly disappear during neap tide on 11 June, coincident with the maximum rate of warming and the onset of bleaching.

Dongsha Atoll is a massive (25 km diameter), circular coral reef emerging from ~500 m water depth on the continental shelf slope in the northern SCS. The living reef flat encircling the lagoon is just 1–3 m deep (Fig. 2d), as is characteristic of many coral atolls, and barrier and fringing reefs worldwide. In summer, water on the shallow reef is heated during the day by solar insolation, but is cooled via advection of offshore water across the reef flat by tidal and wave-driven currents (Fig. 3). Consequently, daily average temperatures on the reef flat typically resemble those of the surrounding open ocean. Indeed, mean temperature recorded by our *in situ* logger on the reef flat during June-July-August (JJA) of 2013–2015 was 29.7 °C, practically identical to that of the surrounding open ocean (29.6 °C in both NOAA-OI and NOAA Coral Reef Watch) (Supplementary Fig. S1). However, in June 2015, an anomalous high-pressure system reduced wind speeds and surface wave height across the northern SCS (Fig. 2b and Supplementary Fig. S3). As a result, current speeds on the reef flat decreased by 40–60% compared to the previous two years for which we have data (Supplementary Fig. S3), disrupting the local heat budget (Fig. 3). For several days, heating from solar insolation exceeded the advective cooling that would otherwise keep the reef flat at open-ocean temperatures, adding up to 4 °C to the relatively modest 2 °C open-ocean anomaly. Reef-flat temperatures peaked in excess of 6 °C above the climatological mean June SST (Figs 2 and 3). We diagnosed the causes of this transient heating event using high-resolution physical measurements and a heat-budget analysis (Fig. 3). The extreme temperature (36 °C) reached in June 2015 was a result of global (El Niño warming superposed upon a global warming trend), regional (high pressure system and reduced winds), and local hydrodynamic (shallow reef, neap tide and unusually slow currents) factors aligning – at the right time – to drive intense heating (see Supplementary Information for additional details).

**Figure 4:**
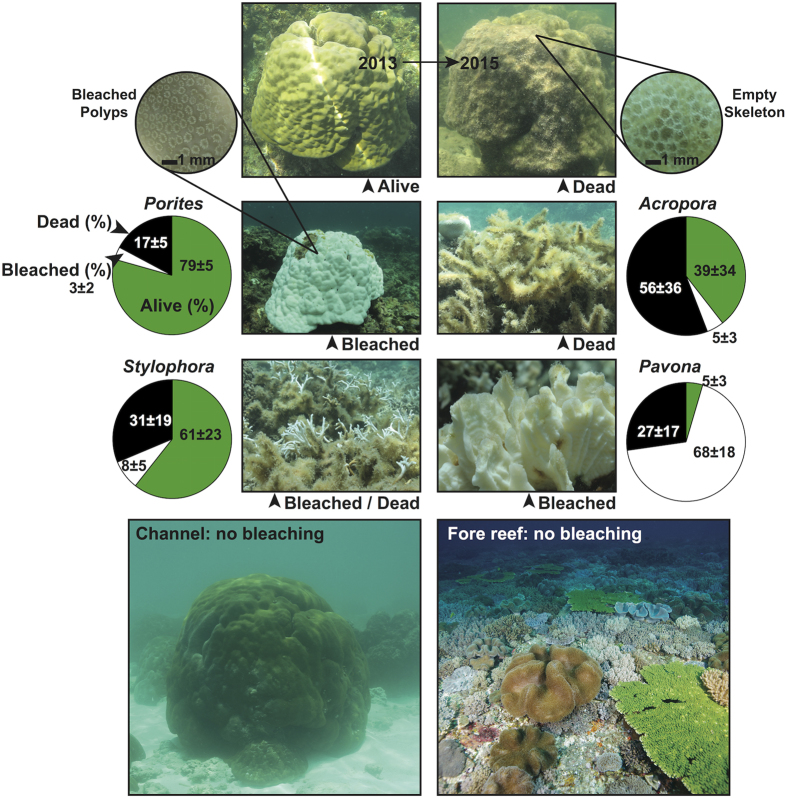
Response of the Dongsha Atoll reef flat coral community to thermal stress. At the top, a 100-year old coral colony photographed alive in June 2013 (left) and after its death in July 2015, confirmed by absence of living polyps (inset). Below, bleaching-induced mortality varied by genus. Green, white, and black areas represent percent of live and healthy/pigmented, bleached, and recently dead corals, respectively, by genus in late July surveys. Live polyps were present in bleached *Porites* colonies (inset), whereas most *Acropora* succumbed quickly and were covered in algae after death. Photos at the bottom show healthy and pigmented corals in the channel and on the upper fore reef slope in late July. The absence of bleaching in the channel was based on visual observations, while ecological surveys were conducted on the fore reef.

Ecological surveys conducted across the reef flat in early June prior to the bleaching event, and again in late July provide a rare quantitative characterization of the response of the benthic community to the extreme thermal stress^7^. In early June, live, healthy corals on the reef flat covered 22% of the benthic area. Mass coral bleaching was observed two weeks later, coincident with maximum temperatures. By late July, bleaching gave way to mass mortality and the cover of live, un-bleached coral was halved to 11% of the benthic area. Our ecological survey point-counts showed 33–40% of coral points recently dead and 10% still bleached (Fig. 4). The response of the benthic community was unusually rapid. Whereas corals typically bleach – and recover – in response to several months of accumulated heating or cooling^28^,^29^, corals on the Dongsha reef flat bleached within one week of peak temperatures and 90% of them were either recovered or dead less than six weeks later.

In contrast to the reef flat, no bleaching was observed on the upper fore reef slope or in the channel north of Dongsha Island, where local amplification of warming did not occur. Large-amplitude internal waves are generated on tidal frequencies in the Luzon Strait to the east of Dongsha Atoll and propagate along the thermocline (70–100 m depth) into the northern SCS^17^,^30^. When these internal waves collide with Dongsha Atoll, they deliver deep, cool water up the fore reef slope^17^,^30^. As a consequence, temperatures at 7 m depth on the fore reef decrease as much as 8 °C for several hours each day^17^, and during 6–15 June 2015, eastern fore reef temperatures were on average 2.8 °C cooler than the surrounding open-ocean SST (Fig. 2e). Yet over the same time in the channel north of Dongsha Island, where the internal waves are absent, mean temperature was within 0.1 °C of the open-ocean SST (Fig. 2e). The lack of bleaching in the channel indicates that the 2 °C open-ocean anomaly was insufficient to drive bleaching on its own and that internal waves may not have been necessary to relieve thermal stress and prevent bleaching on the upper fore-reef slope. Rather, the synergy of global, regional, and local drivers of warming was necessary to drive mass bleaching and mortality on the reef flat.

We observed strong species-specific patterns in mortality as a result of bleaching on the reef flat (Fig. 4). Based on visual surveys conducted on 20 June, all colonies of *Porites, Acropora, Pavona,* and *Stylophora*, the four most common coral genera on the reef flat, appeared bleached. However, six weeks later, we found only 17% mortality of *Porites* in our ecological point-count data, compared with 56% for *Acropora* (Fig. 4). Contrasting bleaching responses have the potential to shift coral reef community composition in favor of the most resistant species^31^,^32^. *Acropora*, though vulnerable to bleaching, has relatively high growth rates and fecundity^32^,^33^,^34^. These traits enable recovery of partially depleted populations, but only over decades and in lieu of reoccurring thermal stress events^32^. Branching corals, including *Acropora*, create unique habitat for many other reef taxa^5^,^35^,^36^. Their selective demise, therefore, creates additional problems for the biodiversity of coral reef ecosystems^32^, which harbor an estimated one quarter of all marine species^37^.

Mass bleaching-induced mortality of long-lived corals across the Dongsha reef flat suggests that an event of this magnitude is unusual, and perhaps unprecedented over the past several decades. Bleaching was reported in the waters immediately surrounding Dongsha Island in 1998 (ref. 38), but the larger lagoon and reef flat were not monitored at that time. To assess whether past high temperature events in the open ocean drove similar levels of ecological response, we examined skeletal cores from massive *Porites* corals for evidence of stress banding, discrete anomalously high-density bands of skeleton accreted during bleaching^39^,^40^,^41^,^42^,^43^. Colonies that bleach but survive and continue to grow, preserve within their skeleton a high-density stress band visible in computerized tomography (CT) scans^39^,^40^,^41^,^42^. Recent evidence suggests that *Porites* stress bands are reliable archives of past bleaching events because their prevalence scales proportionally to community-level bleaching^43^. Partial colony mortality, indicative of severe bleaching, is also visible in CT scans, and reductions in growth due to slow recovery can be quantified from CT images^39^,^40^,^41^,^42^.

**Figure 5:**
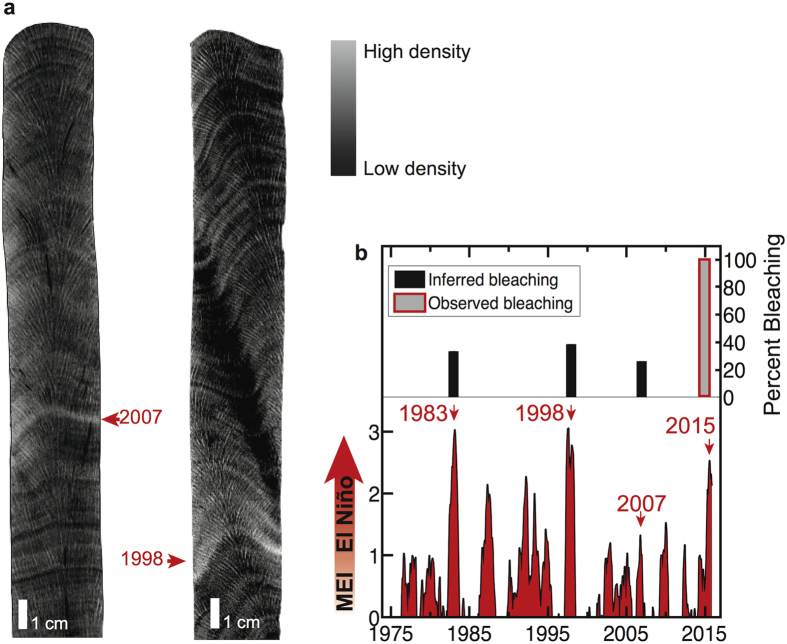
Reconstruction of past bleaching on Dongsha Atoll reveals the 2015 event to be unprecedented in 40 years. (**a**) Computerized tomography (CT) scans of skeletal cores extracted from living colonies show annual growth bands and thin, discrete, anomalously high density bands indicative of bleaching in 1998 and 2007. (**b**) High temperature events associated with El Niño (bottom) caused corals to bleach on Dongsha Atoll, as evidenced by the occurrence of stress bands in 30–40% of *Porites* in 1983, 1998, and 2007 (top). By comparison, 100% of *Porites* colonies bleached in 2015, making this event unprecedented in at least the past 40 years. El Niño events are identified based on the Multivariate ENSO Index (MEI), which quantifies El Niño variability based on a blend of Pacific-wide air pressure, winds, temperatures, and clouds^58^.

We analyzed CT scans of cores from 22 *Porites* colonies on the reef flat ranging in height from 1 to 1.5 m. Each colony was alive and pigmented in early June, prior to the peak temperature anomaly, all appeared bleached by mid-June, and 11 (*i.e.*, 50%) of these colonies died by late July. Average growth rate was 1.5 cm yr^−1^ (Supplemental Fig. S5), indicating that these colonies were 70–100 years old. This means that each colony had survived prior high temperatures associated with El Niño events in 1983, 1998, and 2007 (Figs 1 and 5). However, our analysis of the skeletal records shows that less than 50% of the colonies had bleached during these events, compared with 100% in 2015 (Fig. 5), and there were no signs of partial mortality and no significant declines in annual calcification rate (Supplementary Fig. S5). This implies that the 2015 bleaching event was the most severe to hit Dongsha Atoll in at least the past 40 years, and possibly much longer.

Reef-building corals typically live near the upper limits of their thermal tolerance^7^,^8^. Global climate models project that conditions on the majority of coral reefs will exceed these limits by the second half of this century^10^,^11^. Reducing greenhouse gas emissions in an effort to cap open-ocean warming to 2 °C could delay these impacts and may allow some corals time to acclimate and adapt^10^,^11^,^12^. However, most projections of coral reef futures under a 2 °C global warming scenario rely solely on estimates of open-ocean warming without considering the compounding effects of regional climate and local hydrodynamics. Our results indicate that these projections may be overly optimistic for many shallow coral reef ecosystems.

## Methods

### Climate data

Sea surface temperature (SST) data were acquired from the Extended Reconstructed SST (ERSST) product^44^, NOAA Optimum Interpolation (NOAA-OI)^45^, NOAA Coral Reef Watch^46^, and Moderate Resolution Imaging Spectroradiometer (MODIS)^47^. The different SST products possess a range of temporal coverage and spatial resolution. ERSST covers the entire 20^th^ century at relatively coarse (2° by 2°) resolution, NOAA-OI begins in 1982 at 1° resolution, Coral Reef Watch begins only in 2013 at high resolution (5 km), and MODIS covers only 2002-present but at very high spatial resolution (4 km). ERSST and NOAA-OI anomalies were calculated relative to the 1940–1970 ERSST mean. The NOAA Coral Reef Watch program calculated monthly climatologies for 1985–2012, and we used this as the climatology in our assessment of SST anomalies in the open ocean around Dongsha Atoll during June 2015. ERSST data were used to evaluate the centennial-scale warming trend in the northern South China Sea (Fig. 1). MODIS was used to plot the high-resolution distribution of SST in and around Dongsha Atoll for the eight-day period covering the onset of bleaching (*i.e.* MODIS data were used only to make Fig. 2c). Sea level pressure (SLP) data (2.5° resolution) were acquired from the National Centers for Environmental Prediction/National Center for Atmospheric Research (NCEP/NCAR) Reanalysis^48^. Monthly SLP anomalies were calculated relative to the 1949–2015 monthly climatology. Hourly significant wave height data in the northern SCS from 2010 to 2015 were acquired from a buoy (21.021°N, 118.861°E) maintained by the Taiwan Central Weather Bureau. Sea level variations were calculated following Ray^49^.

### Local reef conditions

The physical conditions leading to the bleaching event were monitored with a series of instruments deployed on Dongsha Island and underwater on the reef flat (Fig. 3). Solar radiation, wind speed, air temperature, precipitation, and relative humidity were measured every 6 minutes on Dongsha Island with a meteorological station maintained by the Dongsha Atoll Research Station. Seawater temperature was monitored on the Dongsha Atoll eastern reef flat (station E5) at 2 m depth, on the eastern fore reef (station E1) at 7 m depth, and in the channel north of Dongsha Island at 5 m depth with Onset Hobo U22 temperature loggers deployed on buoys 0.5 m above the bed (accuracy ± 0.1 °C after calibration in an isothermal bath), sampling every 15 minutes. Currents at station E5 on the reef flat were measured between June 2014 and June 2015 with a Lowell Instruments Tilt Current Meter (TCM; sampling every 5 minutes in 1-minute bursts at 8 Hz) and 16–28 June 2013 and 1–17 June 2014 with Nortek Aquadopp acoustic Doppler current profilers (ADCP; sampling every 4 minutes). The TCM was calibrated to the depth-mean current velocity measured by an ADCP during a 1-week co-deployment in June 2014 at station E5 (r = 0.92, major axis slope = 0.6).

### Heat budget calculations

We used the Dongsha Island meteorological data to estimate the air-sea heat flux in June 2015 as the sum of latent, sensible, longwave radiation, and shortwave radiation fluxes (Fig. 3). Longwave radiation was estimated following Reed (1976)^50^, and latent and sensible fluxes were estimated using bulk-formula calculations with COARE 2.6^51^ following the approach previously developed and validated on Red Sea reefs with similar bathymetry to the Dongsha reef flat^16^. Heat fluxes were converted to temperature (*T*) change by: 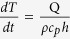, where *t* is time, Q is heat flux in W m^−2^, *ρ* is seawater density (kg m^−3^), *c*_*p*_ is the heat capacity of seawater (W s kg^−1^ °C^−1^), and *h* is water depth (m). The total heat budget (in °C hr^−1^) on the reef flat was determined by taking the time-derivative of measured water temperature, and the advective component was estimated as the difference between total (observed) and air-sea (calculated) components (benthic and diffusive heat fluxes assumed negligible).

### Ecological surveys

Ecological surveys were conducted at seven stations across the reef flat and two stations on the fore reef following a protocol similar to previously established methods for characterizing benthic cover on coral reefs^52^. Pre-bleaching surveys were conducted between 29 May and 7 June^53^, and post-bleaching surveys were conducted between 27 July and 2 August. At each station, 5 × 50 m transects were laid out and photographed every meter (0.5 m by 0.5 m image area), giving a total of 250 photographs per station. Transects were oriented N-S (along-shore) and spaced 5 m apart (cross-shore). Images were analyzed using the program Coral Point Count^54^ with 5 randomly placed points per image identified to coral genera or benthic substrate type (Supplementary Table S1). The same survey methodology was repeated at the same locations pre- and post-bleaching for reef flat stations (E2-E5), while fore reef station E1 was surveyed only post-bleaching. The channel north of Dongsha Island was inspected visually for bleaching on 24 June and 29 July, but no photo surveys were conducted. In total, we made 22,500 point identifications in our study. All corals, whether alive and pigmented, bleached, or recently dead were identified to genera level. Bleached corals were identified based on lack of pigment and the presence of live polyps, whereas recently dead corals were distinguished based on structurally intact corallites without any live polyps present (see also Supplementary Information).

### Bleaching histories and calcification rates

Coral skeletal cores were collected from massive *Porites* colonies using underwater pneumatic drills with 3 cm diameter drill bits. The cores were scanned at Woods Hole Oceanographic Institution Computerized Scanning and Imaging Facility and skeletal density was calculated by comparison to previously calibrated coral skeletal density standards^55^. Annual calcification rates were calculated using the software program coralCT^56^ and the mean calcification rate was calculated for 2007–2012, the years that are overlapping among all colonies^57^. Stress bands were identified visually in 1983 (1/3 cores), 1998 (5/13 cores) and 2007 (6/22 cores) from coral CT scans following previous studies that linked observed bleaching with anomalous high-density band formation^40^,^41^,^42^,^43^.

## Additional Information

**How to cite this article:** DeCarlo, T. M. *et al*. Mass coral mortality under local amplification of 2 °C ocean warming. *Sci. Rep.* **7**, 44586; doi: 10.1038/srep44586 (2017).

**Publisher's note:** Springer Nature remains neutral with regard to jurisdictional claims in published maps and institutional affiliations.

## Supplementary Material

Supplementary Information

Supplementary Dataset 1

Supplementary Dataset 2
