# Supplementary material for: Mass coral mortality under local amplification of 2 °C ocean warming
Source: Sci Rep. 2017 Mar 23;7:44586. doi: 10.1038/srep44586 (PMC5363223; doi:10.1038/srep44586)
Supplement: Supplementary Information [file srep44586-s1.pdf]

Mass coral mortality under local amplification of 2 °C ocean warming

Thomas M. DeCarlo, Anne L. Cohen, George T.F. Wong, Kristen A. Davis, Pat Lohmann,
Keryea Soong
correspondence to: tdecarlo@uwa.edu.au

Supplementary Text

Supplementary Figures S1-5

Supplementary Tables S1-2

Supplementary Text:

Additional details of heat budget analysis and temperature variability

We used temperature and weather data collected on Dongsha Atoll to investigate the driving factors of the 2015 thermal stress event. Our underwater temperature loggers show that reef flat temperatures generally track offshore SST but with strong diurnal and fortnightly variability (Fig. S1). Typically, water temperature on the reef flat is warmer than offshore during the day, and cooler than offshore at night. Fortnightly variability also modulates the daily mean reef flat temperature, occasionally causing multi-day periods when reef flat temperatures are persistently cooler or warmer than offshore (Fig. S1). Much of this fortnightly variability is driven by spring/neap tidal changes in currents, which cool the reef by exchanging reef flat and offshore waters. Associated with most summertime neap tides, when tidal-driven currents are reduced, reef flat temperatures are elevated above the offshore SST for several days. Yet, exceptions to these patterns exist. During June 2015, weak winds and waves associated with an unusual atmospheric high-pressure system in the northern SCS reduced currents speeds by 40-60% compared to June 2013 and June 2014 (Fig. S2-3). As a consequence, daily mean reef flat temperatures were consistently warmer than offshore SST during the entire first half of June 2015, throughout both spring and neap tides (Fig. S1). Intense heating on the reef flat during neap tide is common, however, and certainly not unique to June 2015. For example, neap tide heating events of similar magnitude are evident in June-July 2014 and in May 2015 (Fig. S1). The mid-June 2015 heating is set apart from the previous two years of temperature variability because the neap tide warming was superposed on to already anomalously warm offshore SST, and onto reef temperatures that were already elevated above the offshore SST under anomalously slow currents that persisted for two weeks (Fig. S2).

Ecological surveys

In our ecological surveys, total coral cover (including live, bleached, and dead) was in excellent agreement pre- and post-bleaching for stations E2, E3, E4.5, and E5, but total pre-bleaching coral cover was greater than that observed in post-bleaching surveys for stations E2.5, E3.5, and E4 (Table S1). Thick algal turfs, which quickly overgrow and obscure recently dead corals, make our recently dead coverage a lower-bound estimate (Fig. S4).

Coral calcification rates

We used our 22 *Porites* skeletal cores to assess whether annual calcification rates decreased during previous high temperature years (Table S2). We determined the linear trend of annual calcification rates from 1990-2013 [calcification (g cm^{-2} yr^{-1}) = (1.310 ± 0.031 g cm^{-2} yr^{-1}) + (0.0061 ± 0.0024 g cm^{-2} yr^{-1} per year since 1990)] (± standard error), following the statistical approach of (ref. 57) that accounts for random effects among cores and autocorrelation within the time series. Calcification rates predicted from the linear trend for 1983 (1.27 ± 0.04 g cm^{-2} yr^{-1}), 1998 (1.36 ± 0.04 g cm^{-2} yr^{-1}) and 2007 (1.41 ± 0.05 g cm^{-2} yr^{-1}) were less than, or within uncertainty of, measured calcification rates for those years (1.3 ± 0.3 g cm^{-2} yr^{-1}, 1.49 ± 0.12 g cm^{-2} yr^{-1} and 1.51 ± 0.05 g cm^{-2} yr^{-1}, respectively), indicating that there were no significant declines in calcification during these past bleaching events (Fig. S5).

Supplementary Figures

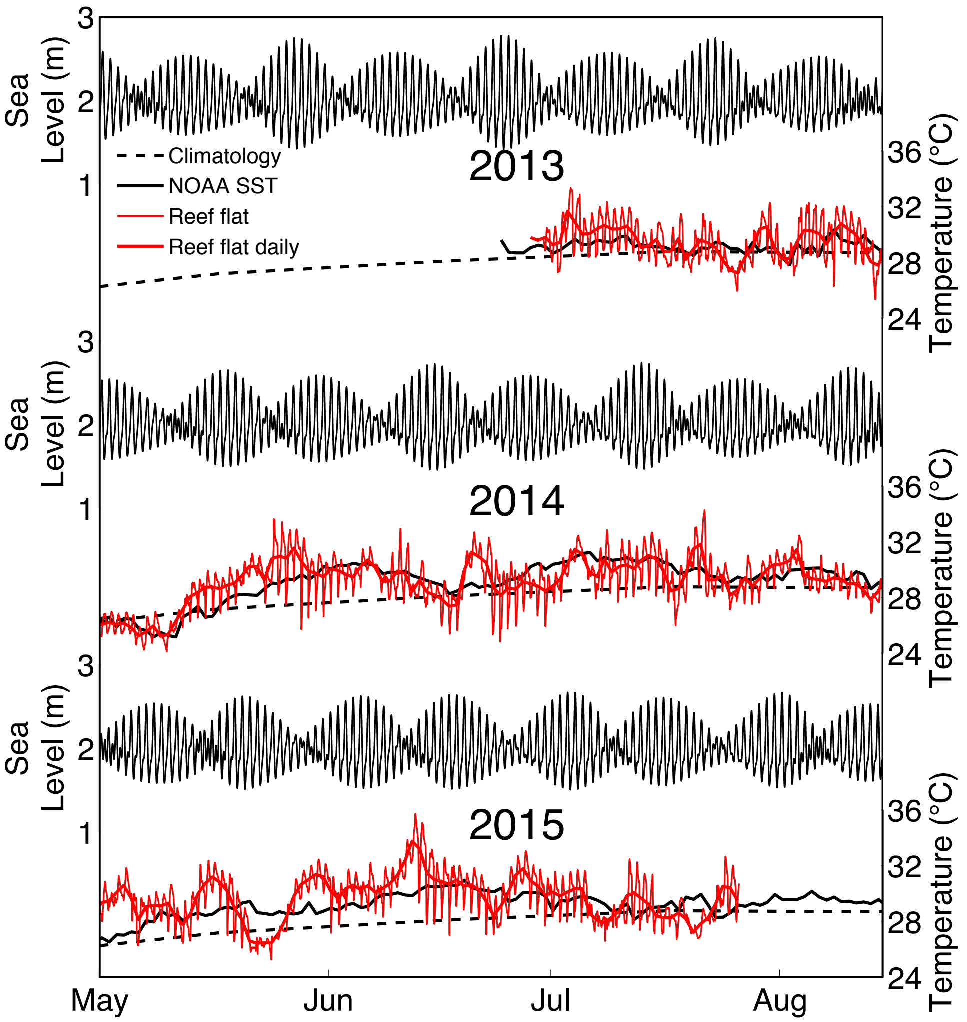

Figure S1. Time series of summertime sea level and water temperature at station E5 on the eastern reef flat. Solid black, solid red, and dashed black plots show sea level, reef flat temperature, and SST climatology, respectively, for years 2013 (top), 2014 (middle), and 2015 (bottom). The climatology (dashed black; NOAA Coral Reef Watch 5-km product monthly means) is the same for all temperature panels, and thus provides a baseline for comparing the reef flat temperatures (red) among years. Sea level fluctuations were estimated with the model of (ref. 49). Reef flat temperatures generally track the open-ocean, but often increase during neap tides (*i.e.* when the sea level amplitude is at a minimum). The greatest temperatures occurred in June 2015, when reef flat temperatures were consistently elevated above the open ocean for several weeks, and were superposed on a 2 °C open-ocean anomaly.

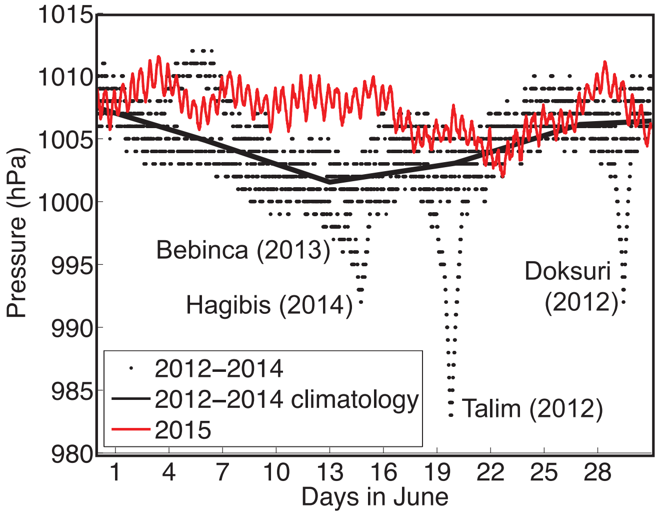

Figure S2. Atmospheric pressure during June 2012-2015 measured at the meteorological station on Dongsha Island. Air pressure in June 2015 (red) was higher than the June 2012-2014 climatology (black line) during almost the entire month. Named tropical storms are indicated next to the associated low-pressure anomalies. The greatest pressure anomaly in June 2015 occurred during 8-15 June, leading to the period of low winds and current speeds, and the onset of bleaching.

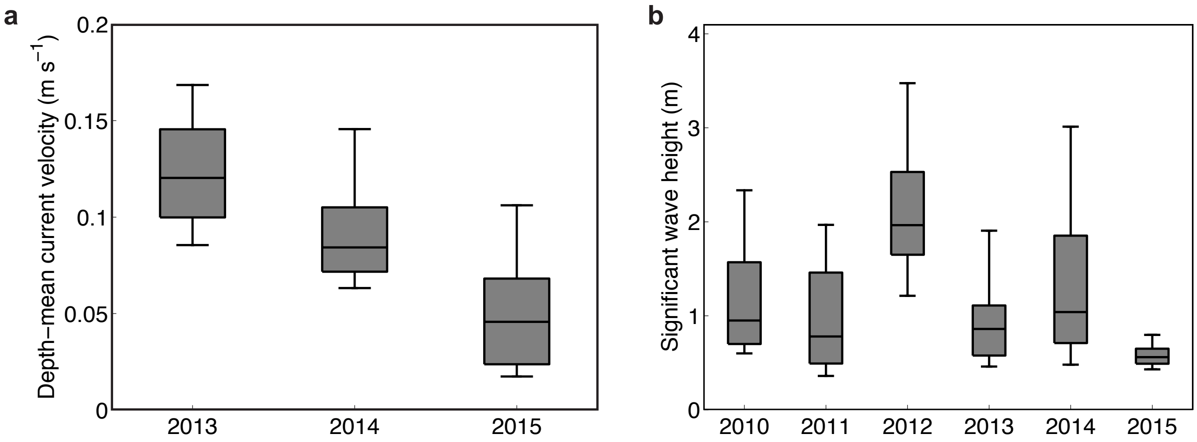

Figure S3. Dongsha reef flat currents and SCS significant wave heights. **a**, Depth-mean current velocities measured at reef flat station E5 in June of 2013, 2014, and 2015. **b**, Significant wave height measured at the Taiwan Central Weather Bureau's "Pratas Buoy" in the northern SCS (21.021°N, 118.861°E) during 1-20 June of each year between 2010-2015. In each box plot, horizontal black lines are medians, gray boxes are the interquartile ranges, and vertical bars are 10th to 90th percentiles.

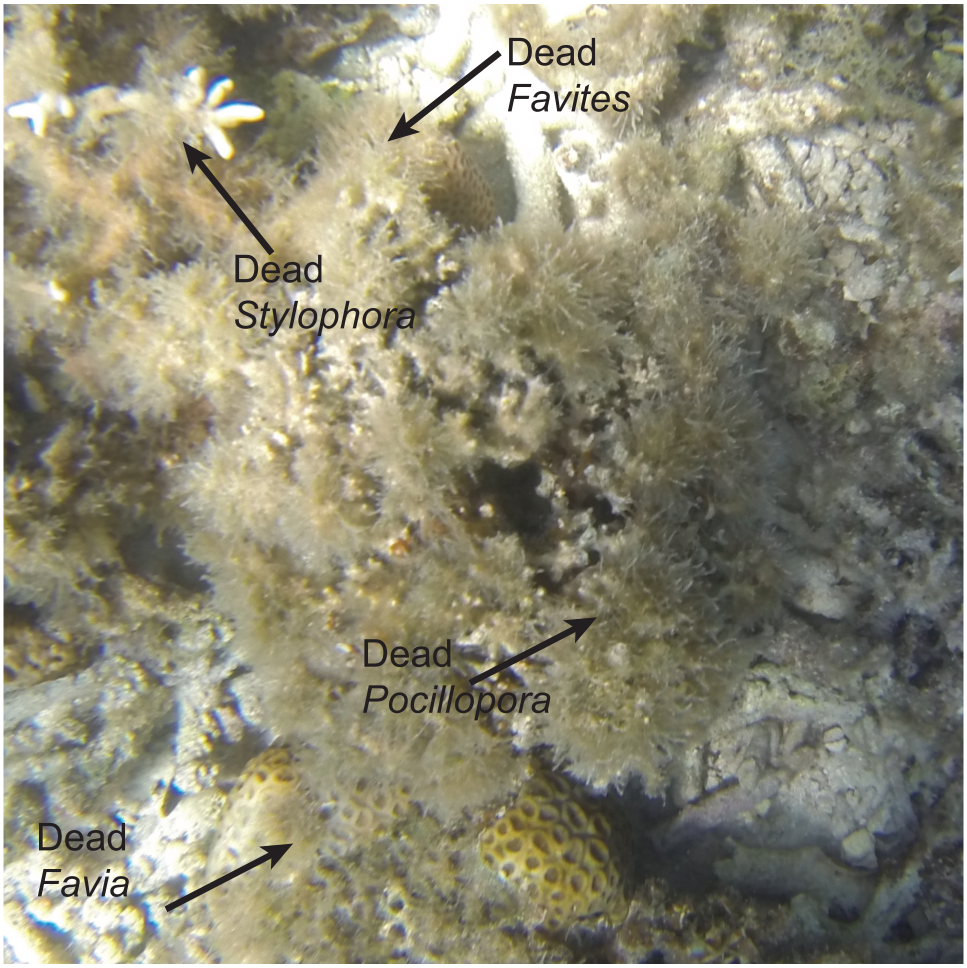

Figure S4. Photograph from the post-bleaching ecological survey at station E5 showing how recently dead corals are partially obscured by algae. Dead or partially dead colonies of *Stylophora*, *Favites*, *Favia*, and *Pocillopora* are all present in this image, all covered with algae. While we are able to identify the recent mortality in this image, we cannot exclude the possibility that algae hid some recently dead coral colonies in post-bleaching surveys.

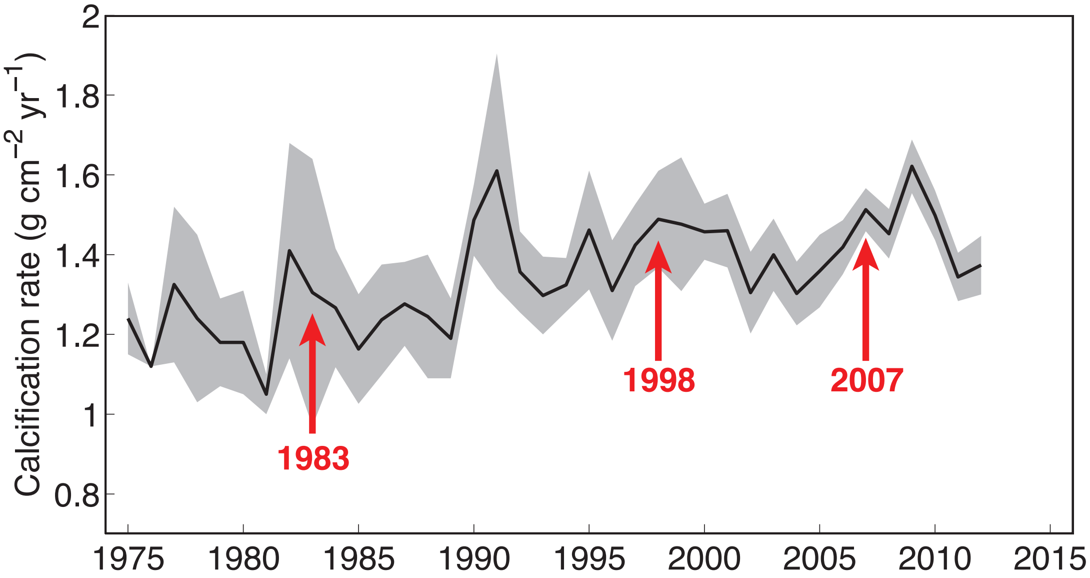

Figure S5. Mean annual calcification rates of *Porites* coral colonies on the Dongsha eastern reef flat. Black line is the mean and gray shading is the standard error among multiple colonies. Calcification rates during bleaching years (1983, 1998 and 2007) were within error of the multi-decadal trend, indicating that annual calcification rates were unaffected by bleaching.

Table S1. Ecological survey point counts. White and shaded rows are pre- (29 May – 7 June) and post- (26 July – 2 August) bleaching surveys, respectively. Each column is a separate survey site on the reef flat (E5 to E2) and on the fore reef (E1). For coral genera rows, each entry is listed as live and pigmented / bleached / recently dead.

Table S2. Coral core calcification and stress band data. Each column is a separate coral colony from station E5, and the first row indicates the core identification number. The second row shows the colony status when surveyed in July 2015. Stress bands are tabulated in the third row, and annual calcification rates are reported in the bottom portion of the table. Empty entries indicate that data were not available for that year, because the core was either off-axis or broken.
